# Use of Instagram by Pre-Service Teacher Education: Smartphone Habits and Dependency Factors

**DOI:** 10.3390/ijerph17114097

**Published:** 2020-06-08

**Authors:** José-María Romero-Rodríguez, Carmen Rodríguez-Jiménez, Magdalena Ramos Navas-Parejo, José-Antonio Marín-Marín, Gerardo Gómez-García

**Affiliations:** Department of Didactics and School Organization, University of Granada, 18071 Granada, Spain; romejo@ugr.es (J.-M.R.-R.); carmenrj@ugr.es (C.R.-J.); magdalena@ugr.es (M.R.N.-P.); jmarin@ugr.es (J.-A.M.-M.)

**Keywords:** smartphone addiction, Instagram, physical education, self-esteem, student welfare, cognitive functions

## Abstract

It is increasingly common to upload photographs on the Internet of sports practices carried out. However, this behavior can be related to smartphone addiction, which has become a global problem. In turn, the intensive use of the Instagram social network has begun to be linked to addictive behaviors on mobile devices. The purposes of this paper were to analyze the Instagram usage habits of future primary school teachers, to determine the influence of sociodemographic factors on intensive Instagram use and smartphone addiction, and to determine the influence of intensive Instagram use on smartphone addiction. For this purpose, a transversal design was adopted where two standardized scales were applied to a sample of university students of the Primary Education Degree of the University of Granada, Spain (*n* = 385). The results showed that the type of image most uploaded to this social network was the selfie, well above sport. Furthermore, the structural equation model highlighted the significant influence of the intensive use of Instagram and smartphone addiction. Finally, the implications and findings of this study are discussed, highlighting the importance of generating healthy habits regarding the use of technology.

## 1. Introduction

The use of social networks is increasingly common among young people. The social network Instagram is one of the social networks with the largest number of users. The ease of access to it and its operation based on the exchange of images has made it a great attraction for the youth population. This has led to it becoming one of the most widely used social networks in the world today by this sector of the population [1,2].

Thus, smartphone addiction has intensified in recent times with the emergence of social networks such as Instagram, since this type of addiction is driven by the personal desire to connect socially [3,4,5,6]. Specifically, Instagram facilitates connectivity through a personal profile subscription system (so-called followers). In this way, an interaction is generated based on putting comments or “likes” the photo uploaded by another user. There are also the “stories” which are images or videos published with a time limit of 24 h.

This has been a great attraction for the youth population, which has led to Instagram’s consolidation as one of the social networks with the largest number of users since its appearance in 2010 [7]. Despite its great appeal, studies have begun to confirm that surfing on Instagram for a long period of time is related to the presence of a depressed mood [8,9]. On the other hand, this attraction that it provokes in young people has generated that people of different ages are somehow forced to use information and communication technologies (ICT) and social networks as a consequence of these needs and challenges of our society.

In recent years, sports blogs, social networks that promote interactive communication, photo sharing through different forums and social networks, YouTube videos, etc. and in particular, on Instagram, there is a latent trend on physical exercise, healthy living, and sports activities, which are broadcast as images or video [10,11,12,13]. This trend has created a certain type of profile in the social network of amateurs or specialists who promote healthy living habits. This has led many people to seek information through specific Instagram profiles, from users who are not necessarily experts on the subject, which can lead to a false expectation since the routine or diet performed is likely not to have the desired effect.

For its part, the use of social networks such as Instagram is becoming increasingly associated with addiction to mobile devices [14,15]. At the same time, some authors highlight the existence of relationships between smartphone addiction and the intensive use of Instagram [16].

This has led to the fact that smartphone addiction has begun to be linked to various physical problems and psychosocial disorders [17,18]. In addition to the intensive use of the Instagram social network, which may be an indicator of increasing addiction to mobile devices. This addiction to the smartphone and, in particular, to certain mobile applications can be educated in advance to prevent these attitudes or to be eradicated and make an appropriate and responsible use of these media. In this way, it is necessary to take into account that social networks can have an educational purpose, since their correct use entails the assimilation of a lot of knowledge and theoretical concepts, as well as the development of practical skills [19,20], as is the case with sports disciplines, physical education, and health. The different applications enable interaction with different physical exercise professionals, as well as official websites of organizations related to this discipline [21].

There are several investigations that show the differences between students from different educational levels when interacting with other colleagues in physical education and sport, and also in the interaction with professionals of these disciplines and teachers [22]. Older people interact to a greater degree to feel confident about sharing information and opinions on social networks, while younger people merely play a passive role and read other people’s publications [23].

Similarly, for over a decade, studies have shown the use of social networks to promote health and care [24,25]. In the field of medicine, health and physical education, Instagram along with other applications have found a niche where health professionals and other groups interact by encouraging and communicating through mobile learning [26,27,28]. These types of platforms serve to share information and “posts” about health problems related to physical activity, about exercise routines and diets recommended by experts, something that increases the motivation of those who visualize this content [29,30,31]. At present, the relevance of Instagram in the lives of young people is very noticeable, so its use within the classrooms connecting their leisure spaces through ICT with elements of their training has many advantages [32].

These advantages include [33]: 

The prolonged use of these applications in their informal setting generates a desire to use them in the formal setting, especially when it comes to highly complex concepts and elements to deal with.

Students highlight how social media postings and user interactions make learning engaging, dynamic, and fun [34].

The platforms available on the Internet and the different applications extend learning, crossing borders, and interacting with learners from other disciplines and countries, combining languages and cultures [35].

Therefore, it was essential in this study to analyze their relationship in a sample of university students, since they are the largest population at risk when using these technologies and these applications in both their leisure and academic contexts [36,37]. Because of this, they were established as objectives of the study: (i) to analyze the Instagram usage habits of future primary school teachers; (ii) to determine the influence of sociodemographic factors on intensive Instagram usage and smartphone addiction; (iii) to determine what influence has excessive use of Instagram in smartphone addiction.

Consequently, research questions were established:

RQ1: What is the typology of photographs that future Primary Education teachers publish on Instagram?

RQ2: Does the following socio-demographic factors: gender, age, work situation, use of Instagram for work, and daily use time of Instagram influence the intensive use of this social network and smartphone addiction?

RQ3: Does intensive use of Instagram influence smartphone addiction?

## 2. Method

A quantitative approach was adopted, with a cross-sectional study design [38]. The approach adopted responds to the objectives of the study, which sought to quantify the responses and describe the reality observed.

### 2.1. Participants and Procedure

The participants of the study were university students of a Degree in Primary Education at the University of Granada (Spain) and users of Instagram. The investigation was conducted on the basis of a convenience sampling design. Thus, the total sample was 385 students. The choice of this sector of the population was advocated, due to their customary use of social networks. In the same way, this group represents the future teachers of the next generations, so it is very important to know their perceptions. The representative sample size was set at 385, as this is the number of representative cases for populations over 1,000,000, with a 95% confidence index and a 5% margin of error. In this case, the student population was less than 1,000,000 (*N* = 2080), so the cut-off at 385 students was timely and representative. The data was collected during the 2019–2020 academic year through the application of an online survey conducted through Google Forms. Specifically, it was composed of 147 men and 238 women, aged between 18 and 40 years (M = 23.85; SD = 5.31). All participants gave their informed consent. At the same time, information was provided to all respondents about the purpose of the study and the anonymous processing of their data. The rest of the sociodemographic data is collected in Table 1.

### 2.2. Measures

#### 2.2.1. Social Media Intensity Scale (SMIS)

The SMIS scale evaluates the intensity of the use of social networks through seven items that have good internal consistency and psychometric properties [39]. In this study, the adaptation of SMIS to Instagram was used [40]. The answers are grouped around a four-level Likert scale, where 1 is ‘strongly disagree’ and 4 is ‘strongly agree’. The scores range from seven to 28 points, with higher scores on the scale indicating a higher level of use and intensity. Adequate reliability was obtained for this study (Cronbach’s a = 0.813). 

#### 2.2.2. Smartphone Addiction Scale (SAS-SV)

To measure smartphone addiction, the SAS-SV scale was used, which gathers 10 items with appropriate psychometric properties to evaluate this construct [41]. It has also been validated in the Spanish context [42]. The response mode is categorized based on a four-level Likert scale, where 1 is ‘totally disagree’ and 4 is ‘totally agree’. Scores range from 10 to 40 points, with higher scores indicating a greater degree of addiction to the smartphone. The reliability of this research was good (Cronbach’s a = 0.82).

### 2.3. Data Analysis

The data were analyzed with the statistical analysis program SPSS and AMOS in version 24. The frequencies and percentages of the type of photographs uploaded to Instagram were calculated. Furthermore, we investigated the possible existence of significant differences between the study population from the T-test for independent samples in the case of the comparison of two groups and the ANOVA test for the comparison of two or more groups. Finally, the hypothesis of multivariate normality through the Mardia coefficient was tested as a previous step to the establishment of the structural equation model (SEM). In the SEM, the relationship of each dependent variable to the Instagram intensive and smartphone addiction constructs was verified, while the influence of intensive Instagram usage on smartphone addiction was also checked.

## 3. Results

**Figure 1:**
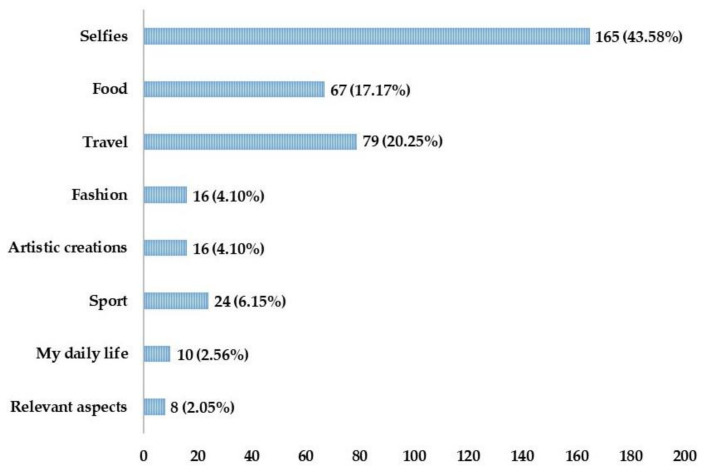
Typology of image publication on Instagram.

In relation to the use of Instagram by future primary school teachers, the main types of images uploaded to the network (Figure 1) are: selfies (43.58%), related to uploading photographs of yourself, where the face and/or body are shown; traveling (20.25%), related to uploading photographs linked to trips made, where the user’s location is highlighted, for example with characteristic photographs of a place, monuments or cultural aspects; and food (17.1%), related to uploading photographs about food, showing dishes cooked by the user himself or the menu ordered in a restaurant. This is followed by sport (6.15%), related to uploading photographs about the sporting activity performed or about some sport practiced by the user; fashion (4.10%), related to uploading fashion pictures, such as store-bought clothes or the recommendation of a particular garment; artistic creations (4.10%), related to uploading photographs about the user’s artistic creations, e.g., paintings, artistic photographs; my daily life (2.56%), related to uploading photographs of what the person does throughout the day, i.e., a minimum of three photographs: one in the morning, one in the afternoon, and one at night. And finally, relevant aspects of my life (2.05%), related to uploading photographs of something important for the user without a temporal sequence, it can be a specific moment in the life of the student but without implying a continuity in time about what they do in each moment, for example awarding a prize, a baptism, a wedding.

In relation to the descriptive data for each scale and according to the socio-demographic data (Table 2), significant differences were only collected in Age in SMIS (*p* ≤ 0.0001) and SAS-SV (*p* = 0.018); Employment situation in SMIS (*p* = 0.002) and SAS-SV (*p* = 0.011); Daily time on Instagram in SMIS (*p* ≤ 0.0001) and SAS-SV (*p* ≤ 0.0001). In the variables where significant differences were obtained, the highest average scores were obtained for intensive Instagram use and smartphone addiction in the ages ranging from 18–21 years, work status as inactive, time of Instagram use of more than three hours.

A value of 0.742 was obtained in the Mardia coefficient. This value was lower than 323, as a result of the formula [p × (p + 2)] [43], where p corresponded to the total of variables observed, in this case, the 17 variables corresponding to the sum of items SMIS and SAS-SV. The confirmation of the hypothesis of multivariate normality allowed the preparation of the SEM. On the other hand, the SEM collected adequate goodness-of-fit indices (Table 3).

Regarding estimates, significant associations were established between age, which was an influential factor in intensive use of Instagram (*p* ≤ 0.0001), age over smartphone addiction (*p* = 0.006), employment situation over smartphone addiction (*p* = 0.019). It was also found that the intensive use of Instagram significantly influenced smartphone addiction (*p* ≤ 0.0001) (Table 4).

**Figure 2:**
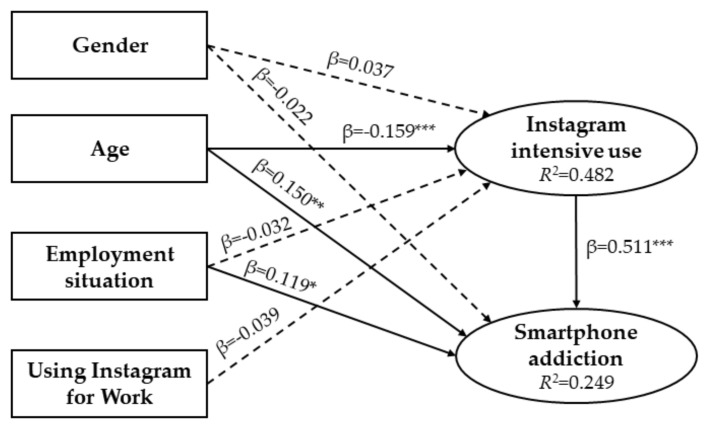
Estimates of the structural equation model Note: β = standardized direct effect; * *p* < 0.05; ** *p* < 0.01; *** *p* < 0.001. Discontinuous arrow = not significant.

In the SEM, the associations of the dependent variables with the two main topics of the study were collected graphically (Figure 2). The construction showed the relation of gender, age, work situation, and use of Instagram by work with the intensive use of Instagram. The link between the intensive use of Instagram and smartphone addiction was also highlighted. It was also of interest to analyze the relationship between gender, age, and work situation with smartphone addiction. The percentage of variation of each construct established by the determination coefficient was 48.2% for intensive use of Instagram (*R^2^* = 0.482) and 24.9% for smartphone addiction (*R*^2^ = 0.249).

## 4. Discussion

Most future teachers, as well as many young people, use Instagram to upload photos of themselves. This could imply concern in the case of compromising photographs. Therefore, it is appropriate that this sector of the population is aware of the problems that these photos may involve in the future, and that they are responsible for uploading images to Instagram [7].

At the same time, food, travel, sport, fashion, artistic creations, personal daily life, and relevant aspects of their lives are other typologies uploaded to the network. They all relate to young people’s need for personal self-expression and use the network to do so [3,4,5,6]. In addition, sporting activity has been reflected as one of the contents that future teachers gather in the social network, above other contents.

The training for the correct use of technology, as well as of social networks, is indispensable so that there are no passive attitudes that progressively become extreme interactions that give rise to addictive behaviors towards social networks [14,15].

In terms of socio-demographic factors, age was a differentiating factor in the intensive use of Instagram and smartphone addiction. Younger university students had a higher degree of use and addiction than the rest, this is related to the type of use they make of the social network according to their age [23]. Furthermore, time of use was a clear indicator of these addictive aspects, where greater use corresponds to a greater degree of addiction and intensive use. On the other hand, the employment situation was another indicator, being a student inactive in work was placed as a risk factor, since the average score was higher. 

The SEM showed the positive and significant influence of the intensive use of Instagram on smartphone addiction. So it was confirmed that there is a close link between these two factors [3,4,15,16]. Likewise, excessive use of Instagram leads to the development of an addiction to the smartphone, which is why it is essential to make regulated use of this social network, as well as others of a similar nature.

Addiction to smartphones and intensive use of social networks are two of the problems that Spanish society must address to improve and prevent health, especially in the young sector. [1,2]. The Government’s efforts to establish preventive measures are limited, although there is increasing attention from public agencies.

However, relating aspects of students’ informal and leisure time environment to physical education, sport, or any health-related activity, through everyday social networks, leads to positive attitudes in students. These attitudes can generate twofold training, on the correct use and development of ICT and all that they imply and on the subject or theme itself. The purpose of this study was to show how action and prevention mechanisms are still necessary for the proper use of social networks since addiction to devices and applications are very common. While addressing the objectives of analyzing the Instagram usage habits of future elementary school teachers and determining the influence of sociodemographic factors and intensive Instagram usage on smartphone addiction. These aspects were key to the trend of using Instagram for promoting physical activity online [10,11,12,13].

## 5. Conclusions

Allowing education to act on this problem is an indispensable question to alleviate the effects that this new paradigm, where technology is framed in the life of students, causes.

Among the limitations of the study is the representativeness of the sample, which can only be generalized to the population of future primary education teachers of the University of Granada. In future studies, it would be advisable to expand the sample at the national level to check whether the data are sustained in larger populations. Additionally, to highlight, the cross-sectional nature of the study, which yields data from a particular time. Therefore, carrying out this study on a longitudinal basis can serve to establish concrete patterns of influence that extend over time. Therefore, in future studies, it would be advisable to replicate it in other contexts to compare the results and check if the incidence patterns are repeated, as well as to extend this type of research over time. However, the value of this study lies in showing current data on the status of future teachers regarding Instagram use and smartphone addiction, which may be generalizable to similar populations. Thus, this study has immediate applicability for developing studies or future studies that investigate this line. On the other hand, it is important to make this data known so that higher education institutions begin to establish concrete measures to alleviate the increase in addiction to smartphones and social networks.

Finally, it is essential to investigate the usage habits of future teachers and educate them on the proper use of technology, since they will be responsible for training future generations.

## Figures and Tables

**Table 1:** Socio-demographic data.

Construct	*n*	%
Gender		
Male	147	38.2
Female	238	61.8
Age		
18–21	123	31.9
22–25	134	34.8
26–29	85	22.1
≥30	43	11.2
Employment situation		
Active	199	51.7
Inactive	186	48.3
Using Instagram for Work		
Yes	45	11.7
No	340	88.3
Daily time on Instagram		
Less than 1 h	118	30.6
Between 1–2 h	146	37.9
Between 2–3 h	68	17.7
More than 3 h	53	13.8

**Table 2:** Descriptive statistical values and significance for each scale based on socio-demographic factors.

Construct	SMIS			SAS-SV		
	M	SD	*p*	M	SD	*p*
Gender						
Male	16.72	4.715	0.912	19.88	5.704	0.943
Female	16.78	4.884		19.92	5.613	
Age						
18–21	18.46	4.511	<0.0001	20.56	5.869	0.018
22–25	17.23	4.234		20.19	5.430	
26–29	14.95	4.606		18.22	4.979	
≥30	13.95	5.451		20.44	6.352	
Employment situation						
Active	16.02	4.621	0.002	19.20	5.758	0.011
Inactive	17.54	4.903		20.66	5.415	
Using Instagram for Work						
Yes	17.89	4.905	0.093	20.71	7.057	0.308
No	16.61	4.789		19.80	5.429	
Daily time on Instagram						
Less than 1 h	12.64	3.932	<0.0001	17.58	4.662	<0.0001
Between 1–2 h	16.69	3.475		19.36	5.040	
Between 2–3 h	19.84	3.137		21.62	5.596	
More than 3 h	22.15	3.060		24.40	6.109	

Note: *p* calculated through the T and ANOVA tests.

**Table 3:** Goodness of fit measure.

Fit Indices	Obtained Values	Criteria
χ^2^	5.434	
*df*	2	
χ^2^/*df*	2.717	≤3
Goodness-of-fit Index (GFI)	0.996	≥0.90
Root Mean Squared Error of Approximation (RMSEA)	0.05	<0.05
Normalised Fit Index (NFI)	0.990	≥0.90
Comparative Fit Index (CFI)	0.994	≥0.90
Adjusted Goodness-of-fit Index (AGFI)	0.944	≥0.90

**Table 4:** Estimates of the parameters of the final model.

Relationship	RW	SE	CR	*p*	SRW
Instagram use ← Gender	0.368	0.380	0.968	0.333	0.037
Instagram use ← Age	−0.774	0.219	−3.541	<0.0001	−0.159
Instagram use ← Employment situation	−0.307	0.410	−0.750	0.453	−0.032
Instagram use ← Using Instagram for Work	−0.587	0.560	−1.049	0.294	−0.039
Smartphone addiction ← Instagram use	0.599	0.055	10.848	<0.0001	0.511
Smartphone addiction ← Gender	−0.250	0.535	−0.467	0.640	−0.022
Smartphone addiction ← Age	0.856	0.312	2.742	0.006	0.150
Smartphone addiction ← Employment situation	1.338	0.571	2.344	0.019	0.119

Note: RW = regression weights; SE = standard error; CR = critical ratio; SRW = Standardized regression weights.
